# Nodular pulmonary amyloidosis diagnosed by ultrasound-guided percutaneous needle biopsy

**DOI:** 10.1016/j.rmcr.2024.102025

**Published:** 2024-04-30

**Authors:** Yuki Ko, Kazunori Tobino, Yuri Hiramatsu, Takuto Sueyasu, Saori Nishizawa, Yuki Yoshimatsu

**Affiliations:** a Department of Respiratory Medicine, Iizuka Hospital, 3-83 Yoshiomachi, Iizuka, Fukuoka, 820-8505, Japan; b Department of Respiratory Medicine, Juntendo University, School of Medicine, 2-1-1 Hongo, Bunkyo-Ku, Tokyo, 113-8421, Japan

**Keywords:** Nodular pulmonary amyloidosis, 18F-fluoro-deoxyglucose (18F-FDG) positron emission tomography, Ultrasound-guided percutaneous needle biopsy, Systemic AL amyloidosis

## Abstract

Pulmonary amyloidosis is characterized by extracellular deposition of fibrous protein called amyloid in the lungs and has three subtypes: nodular, diffuse, and tracheobronchial amyloidosis. Pulmonary nodular amyloidosis can mimic other lung diseases including infectious diseases, metastatic lung tumors, sarcoidosis, and pulmonary hyalinizing granuloma. A biopsy of the lesion is essential for a definitive diagnosis. Herein, we report the case of a 66-year-old man who presented for shortness of breath on exertion and was diagnosed with nodular pulmonary amyloidosis on ultrasound-guided percutaneous needle biopsy. A chest X-ray and computed tomography (CT) revealed bilateral slowly growing multiple calcified pulmonary nodules and cavities. Malignancy was suspected based on 18F-fluoro-deoxyglucose (18F-FDG) positron emission tomography/CT (PET/CT) images. An ultrasound-guided percutaneous needle biopsy was performed, and histopathologic examination of the lesion confirmed nodular pulmonary amyloidosis. This case highlights the importance of considering nodular pulmonary amyloidosis in the differential diagnosis of pulmonary nodules with increased uptake of 18F-FDG on PET/CT and the utility of ultrasound-guided needle biopsy in the definitive diagnosis.

## 1 Introduction

Pulmonary amyloidosis is a disease characterized by the extracellular deposition of amyloid, a fibrous protein, in the lungs [1]. There are three types of pulmonary amyloidosis: nodular pulmonary amyloidosis, diffuse pulmonary amyloidosis, and tracheobronchial amyloidosis [2]. Nodular pulmonary amyloidosis is mostly a localized disease and may resemble other nodular lung diseases such as neoplastic or granulomatous diseases. Therefore, the diagnosis of pulmonary amyloidosis is mainly confirmed by transbronchial lung biopsy, surgical lung biopsy, and autopsy, but there are few reports of diagnosis by ultrasound (US)-guided percutaneous needle biopsy. In addition, 18F-fluoro-2-deoxy-d-glucose positron emission tomography (18F-FDG PET) is mainly used for the diagnosis and staging of malignant tumors, although there are a few reports of 18F-FDG accumulation in lesions in amyloidosis as well [[2], [3], [4]]. Here we present a rare case of nodular pulmonary amyloidosis with high accumulation of 18F-FDG, histologically confirmed by US-guided percutaneous needle biopsy.

## 2 Case presentation

A 66-year-old Japanese man presented to our department with progressive shortness of breath and cough for five years. He denied having any other symptoms, including chest pain, weight loss, fevers, and chills. He has been smoking for over 30 years and has only occasionally drunk alcohol. His medical history included hypertension. His initial vital signs were within normal limits. Physical examination revealed digital clubbing and no rales were heard on chest auscultation. Laboratory tests on admission revealed white blood cell count, 7120/μL; haemoglobin concentration, 14.0 g/dL; platelet count, 31.4 × 103/μL; C-reactive protein concentration, 0.55 mg/dL; IgG, 1, 038 mg/dL; IgA, 1534 mg/dL; and IgM, 20 mg/dL. The serum immunofixation test showed normal free kappa light chain level (17.8 mg/L) and an elevated free lambda light chain level (154 mg/L). The tumor markers, including ProGRP, CEA, and SCC, were within normal limits. The complete blood count, serum electrolytes, renal and liver function, and comprehensive metabolic profile findings were normal. Sputum smears and cultures were negative for acid-fast bacilli, fungi, or other microorganisms. An arterial blood gas analysis obtained while breathing room air revealed PO_2_ of 88.8 mm Hg, PCO_2_ of 42.6 mm Hg, and pH of 7.399. The electrocardiogram revealed atrial fibrillation. The echocardiogram showed normal cardiac function without any abnormal findings.

**Fig. 1:**
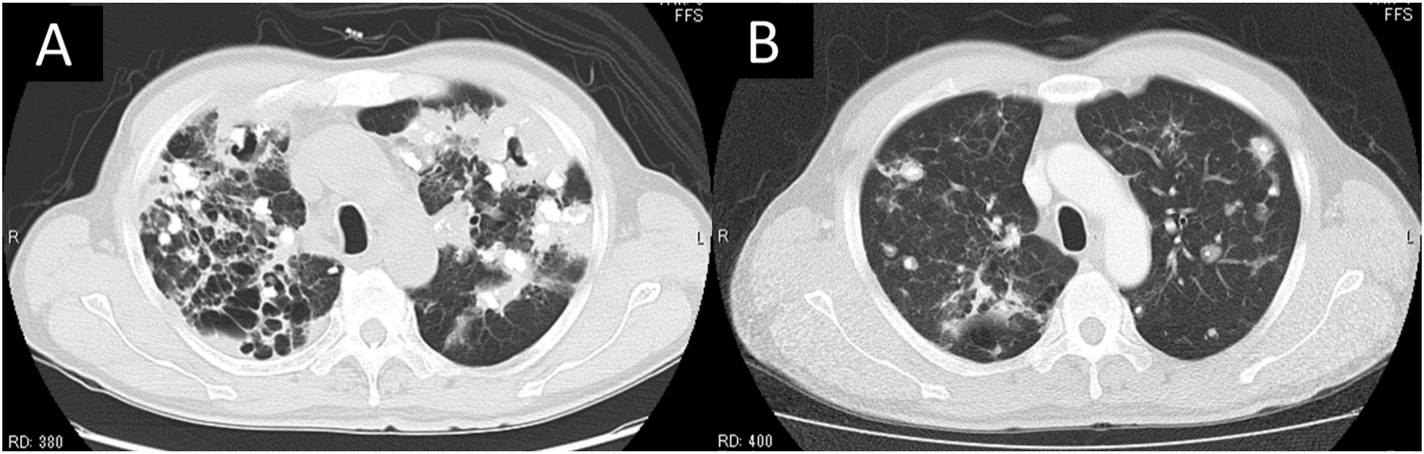
Chest CT imaging on admission showed multiple nodules and masses with calcification and cysts in both lungs (A). A CT scan of the chest 10 years earlier (B) already showed the presence of calcified nodules, which were smaller than on the present CT images.

**Fig. 2:**
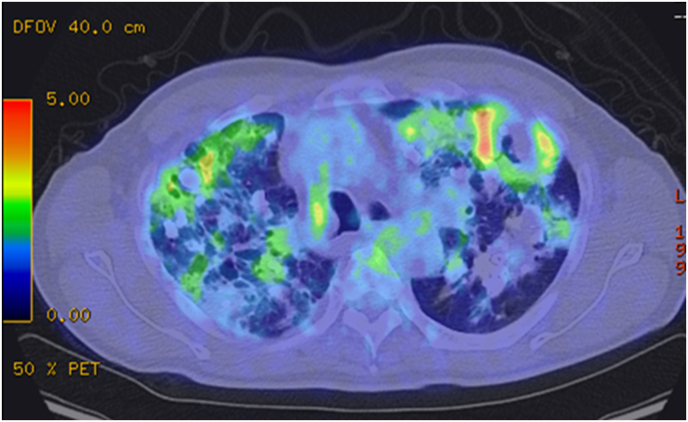
18F-FDG PET/CT showed varying degrees of FDG uptake in non-calcified areas of several nodes.

**Fig. 3:**
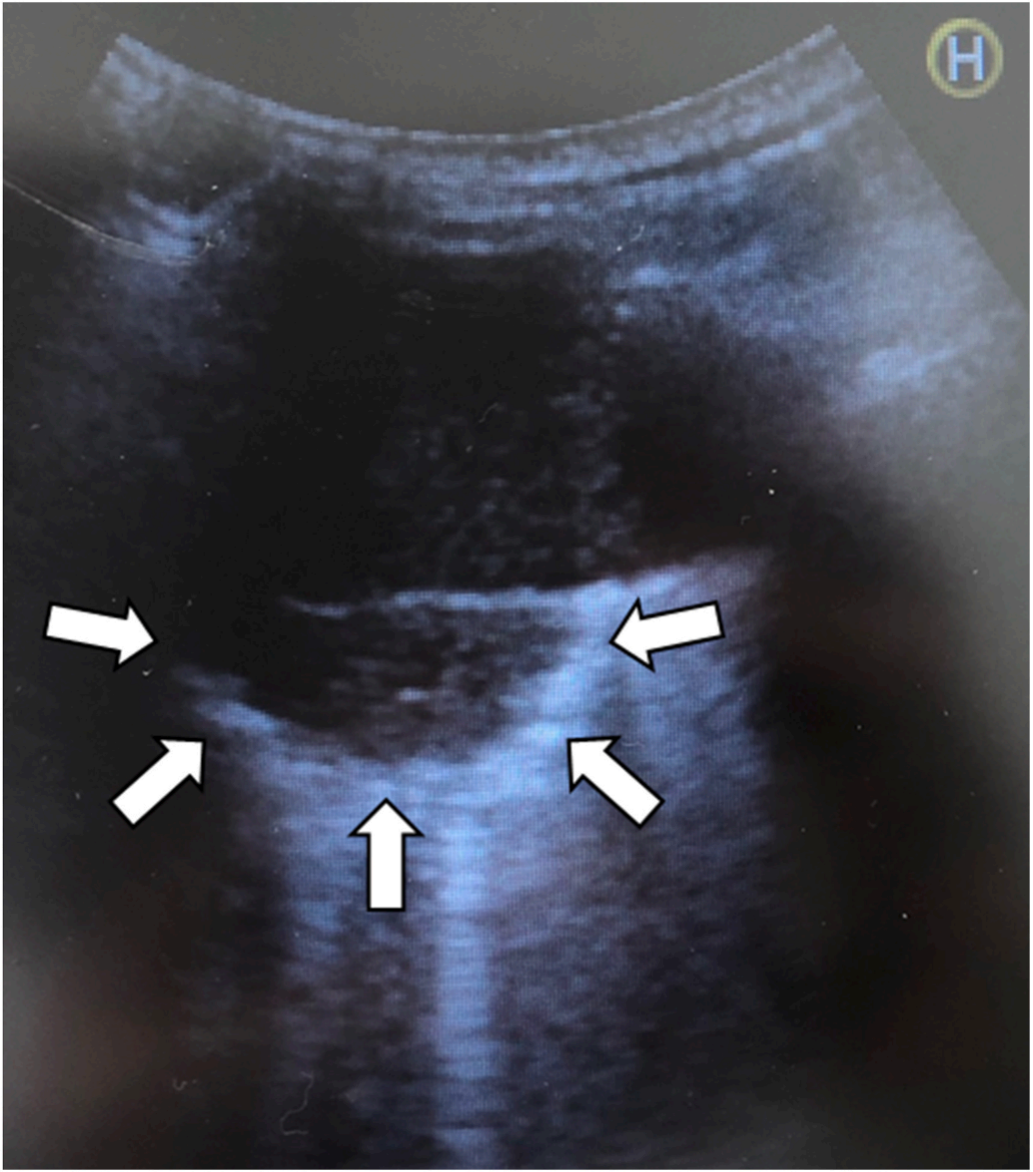
Transthoracic ultrasonography revealed a lesion in contact with the pleura of the left lung (white arrows).

**Fig. 4:**
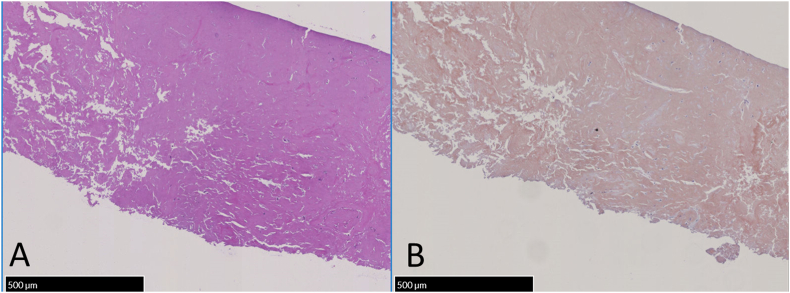
Histopathological findings of pulmonary nodules collected by US-guided biopsy are shown. Hematoxylin-eosin staining (A) shows abundant amorphous eosinophilic material with focal calcification displacing the lung parenchyma. Congo red staining (B) shows that these amorphous eosinophils are pink or red, suggesting amyloidosis.

Chest radiography displayed multiple irregularly shaped calcified nodules predominantly localized bilaterally in the upper and middle lung fields. Chest computed tomography (CT) revealed multiple bilateral pulmonary nodules varying in size up to 5 cm, with no evidence of lymphadenopathy. The largest nodule, measuring 5.2 × 3.8 cm, was noticed in the posterior segment of the left upper lobe. Some nodules were calcified, some cavitated and accompanied by cystic changes around them (Fig. 1A). Mediastinal lymphadenopathy was not detected. Comparative analysis of previous CT images revealed a notable escalation in the number and size of nodules over the last decade (Fig. 1A and B). Given the suspicion of malignancy, 18F-FDG PET/CT was conducted, revealing markedly heightened 18F-FDG uptake in the largest nodule in the left upper lung lobe (maximum standard uptake value, 5.7) (Fig. 2). The remaining lung nodules showed varying degrees of 18F-FDG uptake, lower than those in the left upper lung lobe. There was no 18F-FDG uptake in other organs or bones, suggesting metastasis. Chest ultrasonography revealed a mass in the left lung, located in the left anterior thoracic region and in contact with the pleura, measuring 5.2 × 4.0 cm. (Fig. 3). The lesion exhibited respiratory mobility, was considered to have no pleural involvement, and showed no internal blood flow. The lesion underwent ultrasound-guided transthoracic true-cut needle biopsy using a 19-gauge needle. The biopsy procedure was repeated twice to ensure adequate sampling and accuracy of results and was completed without complications. Histopathologic analysis of the biopsy specimen revealed nodular amyloid deposits in the alveolar space with no evidence of malignant cells or microorganisms. (Fig. 4). Immunofluorescent staining revealed a diagnosis of light chain amyloidosis (AL amyloidosis with IgA and lambda). The patient was then evaluated for evidence of myeloma or plasma cell dyscrasia, and all subsequent tests, including serum and urine protein electrophoresis and immunofixation, bone marrow biopsy, and immunohistochemistry, were normal. Based on these test results, the patient was diagnosed with nodular pulmonary amyloidosis. Low lung function and extensive lung lesions made surgery difficult, and he refused systemic treatment.

Two months after diagnosis, the patient was urgently admitted to the hospital for acute heart failure. Because the patient also presented with type 2 respiratory failure, home oxygen therapy and home non-invasive positive pressure ventilation were introduced, and he was discharged from the hospital. Four years after diagnosis, the patient is still alive despite repeated hospitalizations.

## 3 Discussion

Amyloidosis exhibits a global incidence of approximately 10 cases per million patients yearly [5]. Histologically, amyloid deposits manifest as eosinophilic amorphous deposits, identifiable through Congo red staining and typically distinctive apple-green birefringence under polarized light [3,6]. The common forms of amyloidosis include systemic AL amyloidosis (formerly primary amyloidosis), systemic AA amyloidosis (formerly secondary amyloidosis), systemic wild-type ATTR amyloidosis (formerly age-related or senile systemic amyloidosis), systemic hereditary ATTR amyloidosis (formerly familial amyloid polyneuropathy), and localized AL amyloidosis [[6], [7], [8]]. The localized AL amyloid differs from its systemic counterpart by the morphological appearance of the amyloid and the presence of clonal plasma cells and giant cells. The ƙ light chains in localized form are more frequent than the λ chains, in contrast to the systemic form [9]. Lung involvement is relatively common but typically asymptomatic, unless the amyloid deposits severely affect gas exchange [[10], [11], [12]]. From the pathologists’ perspective, amyloidosis can appear in the lungs in 3 distinct forms: diffuse alveolar-septal amyloidosis, nodular pulmonary amyloidosis, and tracheobronchial amyloidosis [2,6,13].

Nodular pulmonary amyloid deposits are typically found in multiple sites in lungs, usually consisting of immunoglobulin light chain deposits or mixed light chain/heavy chain ones [9,14]. While most patients are asymptomatic, some cases may experience symptoms such as wheezing, stridor, coughing, and recurring pneumonia [15]. Amyloid nodules are often located in the lower lobes, in the peripheral and subpleural areas [5,13]. Multiple pulmonary nodules are common and may exhibit smooth, lobulated, or spiculated margins [13]. Approximately half of the cases show calcification within the nodules, which can have central or irregular pattern [13,16,17]. They can range in size from 0.5 to 15 cm [16,17]. In our case, the largest tumor size was 6.1 cm (Fig. 1). The natural history of nodular pulmonary amyloidosis is benign, with lesions potentially growing in size and number over time, but a good overall prognosis and little need for treatment [13,17]. Cavitation is a rare occurrence [18], but our patient presented with multiple partially calcified and cavitated amyloid nodules in both lungs on CT scan (Fig. 1). Differential diagnoses include primary or metastatic tumors, tuberculosis, silicosis, sarcoidoisis, hamartomas, or pulmonary hyalinizing granuloma [16,17,19,20]. Because of varied imaging appearance, diagnosis through image evaluation alone is difficult. The diagnosis of amyloidosis is predominantly confirmed through transbronchial lung biopsy, surgical lung biopsy, or autopsy. Only one case of pulmonary amyloidosis diagnosed by US-guided lung biopsy has been reported by Mihalova et al. [21].

Although 18F-FDG PET scans play an important role in the evaluation of patients with lung malignancies, 18F-FDG can accumulate in lesions even in nonneoplastic disease [3,4,22]. Various metabolically active nonneoplastic diseases, such as histoplasmosis, sarcoidosis, tuberculosis, and aspergillosis, have been reported to increase the accumulation of 18F-FDG [2,22]. Several case reports have described the role of 18F-FDG PET/CT in the evaluation of AL amyloidosis. A recently published study by Glaudemans et al. reported that increased 18F-FDG uptake has been reported to be present in all five patients with localized AL amyloidosis [23]. Lee et al. showed that 18F-FDG uptake was observed in 11/17 cases (64.7 %) and which included both systemic and localized pulmonary AL amyloidosis [24]. In these previous reports, the standardized uptake value (SUV) value of 18F-FDG in pulmonary amyloidosis lesions was reported to be around 2 to 7, and the value in our patient was 5.7, similar to previous reports. The accumulation of 18F-FDG in amyloidosis is thought to be due to the binding of inflammatory cells to amyloid deposits and the strong uptake of 18F-FDG [25,26]. It has been reported that 18F-FDG uptake is enhanced in neutrophils, macrophages, and lymphocytes activated by cytokines and lipopolysaccharide (LPS), and 18F-FDG accumulation may be enhanced in lesions with a high infiltration of activated inflammatory cells [[27], [28], [29]]. The giant cells in localized amyloidosis may contribute to the formation of the soluble full-length light chains into insoluble fibrils [30]. The high amounts of serum amyloid P make the amyloid deposits unavailable for inflammatory phagocytic cells in systemic amyloidosis [31]. This observation suggests that the differential uptake of 18F-FDG between localized and systemic amyloidosis may reflect variations in the degree of inflammatory cell infiltration. Another two studies reported by Baqir et al. [12,32] showed that mucosa-associated lymphoid tissue lymphoma was associated with pulmonary amyloid and 18F-FDG uptake, which might be due to plasma cell differentiation [33]. Unfortunately, in our patient, histological examination of the biopsy specimen showed no inflammatory cellular infiltrate that would cause high accumulation on 18F-FDG-PET (Fig. 2, Fig. 4). We believe that there is an infiltration of inflammatory cells in the lesion sites that could not be sampled this time.

US-guided lung biopsy offers several clinical advantages. First, both CT- and US-guided procedures demonstrate high accuracy for diagnosing pulmonary lesions [34,35]. Second, compared to CT-guided biopsies, US-guided procedures do not involve radiation exposure and have fewer complications, such as pneumothorax and hemorrhage, attributed to reduced procedural time and real-time guidance [34,35]. Furthermore, the portable US device can be moved to the patient's bedside, allowing the procedure to be performed at the bedside of patients in a variety of positions, such as the semi-recumbent decubitus position for patients with respiratory distress who cannot tolerate the prone abdominal or spinal positions [35]. To the best of our knowledge, only one clinical case of pulmonary nodular amyloidosis confirmed histologically by US-guided transthoracic needle biopsy has been reported, and our patient is the second case [21].

Although cardiac amyloidosis should be considered in patients with acute heart failure, we believe that the likelihood of this condition in our case is low. The patient refused to undergo a myocardial biopsy, which precluded a definitive diagnosis. Furthermore, the patient's heart failure has remained stable over the four years following the initial onset of symptoms, which is not typical of the progressive nature of cardiac amyloidosis. Lastly, as reported by Baumgart et al., localized pulmonary amyloidosis accounts for more than 90 % of amyloidosis cases and is far more common than systemic amyloidosis [36]. Given these factors, we propose that our patient's presentation is more likely attributable to localized pulmonary amyloidosis rather than cardiac amyloidosis.

In conclusion, we have reported a case of multiple pulmonary amyloid nodules with 18F-FDG uptake that acquired via US-guided percutaneous needle biopsy. Pulmonary amyloidosis should be added to the differential diagnosis for cases of multiple pulmonary nodules that show 18F-FDG uptake. It is conceivable that inflammatory reactions caused by amyloid lesions can similarly result in increased 18F-FDG uptake. US-guided lung biopsy is also useful and safe for the diagnosis of pulmonary amyloidosis.

## Patient consent for publication

Written, informed consent was obtained from the patient.

## CRediT authorship contribution statement

**Yuki Ko:** Writing – review & editing, Writing – original draft, Visualization, Data curation, Conceptualization. **Kazunori Tobino:** Writing – review & editing, Visualization, Project administration. **Yuri Hiramatsu:** Writing – review & editing, Data curation. **Takuto Sueyasu:** Writing – review & editing, Data curation. **Saori Nishizawa:** Writing – review & editing, Data curation. **Yuki Yoshimatsu:** Writing – review & editing, Visualization.

## Declaration of competing interest

The authors declare that they have no known competing financial interests or personal relationships that could have appeared to influence the work reported in this paper.

No conflicts of interest.
